# Heat-stability study of various insulin types in tropical temperature conditions: New insights towards improving diabetes care

**DOI:** 10.1371/journal.pone.0245372

**Published:** 2021-02-03

**Authors:** Beatrice Kaufmann, Philippa Boulle, Flavien Berthou, Margot Fournier, David Beran, Iza Ciglenecki, Malcolm Townsend, Guillaume Schmidt, Maya Shah, Susanna Cristofani, Philippe Cavailler, Michelangelo Foti, Leonardo Scapozza

**Affiliations:** 1 Pharmaceutical Biochemistry, School of Pharmaceutical Sciences, University of Geneva, Geneva, Switzerland; 2 Médecins Sans Frontières Switzerland, Geneva, Switzerland; 3 Faculty of Medicine, Department of Cell Physiology and Metabolism, University of Geneva, Geneva, Switzerland; 4 Division of Tropical and Humanitarian Medicine, University of Geneva and Geneva University Hospitals, Geneva, Switzerland; The Ohio State University, UNITED STATES

## Abstract

Strict storage recommendations for insulin are difficult to follow in hot tropical regions and even more challenging in conflict and humanitarian emergency settings, adding an extra burden to the management of people with diabetes. According to pharmacopeia unopened insulin vials must be stored in a refrigerator (2–8°C), while storage at ambient temperature (25–30°C) is usually permitted for the 4-week usage period during treatment. In the present work we address a critical question towards improving diabetes care in resource poor settings, namely whether insulin is stable and retains biological activity in tropical temperatures during a 4-week treatment period. To answer this question, temperature fluctuations were measured in Dagahaley refugee camp (Northern Kenya) using log tag recorders. Oscillating temperatures between 25 and 37°C were observed. Insulin heat stability was assessed under these specific temperatures which were precisely reproduced in the laboratory. Different commercialized formulations of insulin were quantified weekly by high performance liquid chromatography and the results showed perfect conformity to pharmacopeia guidelines, thus confirming stability over the assessment period (four weeks). Monitoring the 3D-structure of the tested insulin by circular dichroism confirmed that insulin monomer conformation did not undergo significant modifications. The measure of insulin efficiency on insulin receptor (IR) and Akt phosphorylation in hepatic cells indicated that insulin bioactivity of the samples stored at oscillating temperature during the usage period is identical to that of the samples maintained at 2–8°C. Taken together, these results indicate that insulin can be stored at such oscillating ambient temperatures for the usual four–week period of use. This enables the barrier of cold storage during use to be removed, thereby opening up the perspective for easier management of diabetes in humanitarian contexts and resource poor settings.

## Introduction

Diabetes is a worldwide health issue, with the affected population estimated to reach 592 million people in 2035, representing a 55% increase over the period 2013–2035 [1]. This increase will be highest in low- and middle-income countries. Whereas most of this burden relates to type 2 diabetes, global disease burden data for type 1 diabetes is lacking. It is estimated that almost half a million children aged 0–14 have type 1 diabetes and that its prevalence is increasing by 3% per year [1]. Injection of insulin is essential to the survival of people with type 1 diabetes, and key to the better management of an estimated 30.2 million people with type 2 diabetes in 2018 [2]. Insulin was discovered in 1921, first administered to a patient in 1922 and included on the World Health Organization’s Model List of Essential Medicines. However, access to this medicine is still problematic in many settings globally and its cost represents a large financial burden on individuals and health systems [3,4]. So far, most of the efforts to improve availability have been targeted at cost reduction and increased local availability of insulin [5].

Manufacturers’ specifications require intact vials of insulin to be stored at low temperatures (between 2 and 8°C, see S1 Table in additional material section), which require regular access to a refrigerator. This is a barrier for diabetes patients in contexts where refrigeration is not always available, affordable, or reliable due to irregular electricity supply. Once a vial has been opened, insulin can be stored at ambient temperature during the usual four- to six-week period of use, depending on the brand and type of insulin. For some formulations product inserts recommend avoiding refrigeration during the period of use. According to manufacturers’ specifications, the maximum ambient temperature should not exceed 25 to 30°C during this period (see S1 Table). However, in tropical regions ambient temperatures often exceed these limit values. Grajower [6] recommends a period of use of 28 days after the first puncture in an intact vial (solutions and suspensions). Insulin in cartridges may undergo more extreme conditions in terms of agitation and temperature variation than insulin in vials and the recommended period of use varies from 28 days for solution down to 10 days for suspensions [7]. Ten years later, there is still no consensus on this issue, although it could prevent unused insulin being unnecessarily discarded [8,9].

Banting et al., the team who discovered insulin, noticed a total inactivation of insulin provoked by exposure to heat [10]. From the literature, insulin without additives exhibits a clear transition from folded to unfolded structure beginning around 50°C, with the transition midpoint (also called melting temperature) at about 65°C, and the Zn-induced hexamer of insulin shows the same transition around 84° [11–13], which suggests the hexameric state of insulin found in the formulations to be relatively stable.

The principal mechanisms of insulin degradation involve both physical and chemical changes that occur at elevated temperatures [14]. Physical degradation is irreversible and leads to fibril formation. Chemical degradation implies changes in the structure of the protein leading to the formation of covalent polymers. It has been documented that insulin’s degradation increases with rising temperatures, particularly those continuously above 30°C [14].

Insulin’s shelf-life at 25°C (isothermal studies [15],) has been estimated at 199.1 days [16], and comparisons between isothermal and non-isothermal experiments have been published. Mechanical agitation combined with heating at 37°C was also investigated [17], with the assessment period limited to seven days. Insulin retained acceptable values in terms of insulin quantification and polymeric forms occurrence. A 14-day study of the chemical stability of insulin solution continuously shaken at 37°C showed a maintained potency [18]. A recent study assessed the stability of commercial insulin formulations [19] over 28 days, combining agitation and heat stress testing at two 10-hour cycles of 25 and 37°C. Active insulin was quantified by HPLC-UV and visual modifications were investigated. In visually changed samples (formation of agglomerates), a greater variability in dose delivery was found. Therefore it has been recommended to discard any insulin formulation that appears visually modified [19].

In 1987, insulin alterability under African climatic conditions was investigated [20]. Insulins available at that time were exposed to storage conditions considered inadequate: ambient temperatures (25 to 34°C) for 60 days. Biological activity of these samples, measured using hypoglycemic testing on mice and liquid chromatographic quantification, was found to be identical to that of reference samples stored in a refrigerator. Unfortunately, no continuous temperature monitoring was done in this study. In line with the intrinsic stability of insulin shown by the relatively high melting temperature values, the protein [11] appears stable in conditions outside the recommendations, and the common practice of systematically discarding any insulin vial that has been exposed to heat may be reevaluated. Vimalavathini et al. studied the stability of insulin exposed to the highest temperatures commonly encountered in India [21] over a period of 28 days. For the higher temperatures investigated (isothermal studies at 32 and 37°C), they determined a decrease in total insulin content of 14 and 18%, respectively. Insulin stored in mud pots (mean temperature 26°C) retained a similar potency to that observed in previous studies at an ambient temperature of 25°C. The authors recommend a period of use reduced to two weeks only when storage cannot be assured at cool temperatures. To address this challenge, Gill and colleagues suggested that some non-refrigerated methods of storage, such as holes in the ground and porous clay pots filled with sand and water could provide possible solutions [22]. A comparison between various traditional cooling devices [23] indicated that this approach may allow safe storage of insulin outside a refrigerator. Besides recommendations on temperature the manufacturers insist on avoiding direct sunlight exposure and freezing.

Despite the presence of several insulin thermostability studies, and although high ambient temperature is a key aspect of reduced insulin accessibility in tropical regions such as sub-Saharan African countries or India, no one to date has addressed the issue of insulin stability related to home storage conditions in tropical regions, characterized by continuously fluctuating temperatures. In this work, therefore, the question addressed is whether commercially available insulin is stable under high oscillating ambient temperatures for a period of four weeks (28 days), which corresponds to the usual recommended period of use of most commercially available insulins upon opening of the vial or cartridge.

## Material and methods

### Materials

#### Studied insulin formulations

Besides human insulin formulations used on the field by MSF (rapid, NPH/isophane and pre-mixed rapid/NPH insulins (mixed)), four other analogue insulin formulations commercially available in Switzerland and France were bought in a local pharmacy and tested, with proper storage conditions ensured (S1 Table in additional material section). Concerning the vials used on the field, the cold chain was respected and monitored (according to usual MSF cold-chain standards) throughout the supply process until reception in the refugee camp pharmacy and throughout its storage there. Therefore, all vials at T = 0 are assumed to be amyloid seed-free.

### Study design

**Fig 1:**
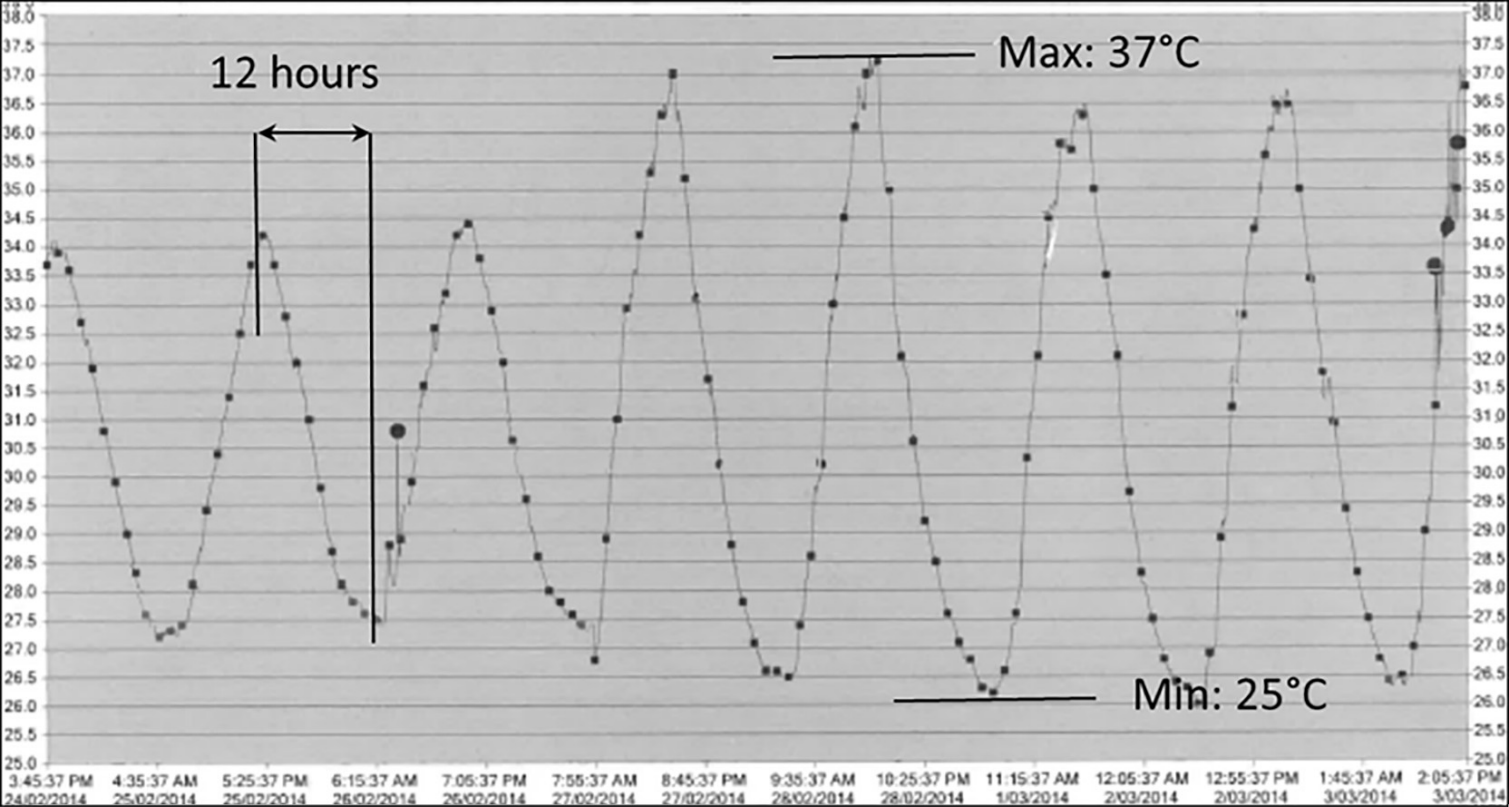
Field ambient temperature measurement over a period of seven days at a patient’s home using log tag monitoring (data provided by MSF).

In Dagahaley refugee camp in northern Kenya, a number of people with diabetes require insulin treatment, but availability of refrigeration at patients’ homes is very limited. The measured ambient temperature oscillated between 25 and 37 ^o^C with a 12-hour cycle, exceeding usual recommendations for insulin use out of refrigeration (Fig 1).

In order to assess insulin stability, the temperature oscillations that were observed in the field were replicated in the laboratory (see S1 Fig in Additional material section and Fig 1), and insulin formulations were exposed to these fluctuating temperatures in the laboratory. A series of mixed insulin vials were stored at ambient temperature in Kenya during a period of 4 weeks of use by patients and brought back to Geneva for analysis. Insulin stored at 2–8°C was used as positive control and heat-degraded insulin as negative control. Furthermore, the effect of continuous exposure to high temperatures of 31 and 37° C was also investigated, as controls comparative with data available in the literature (potency determination).

Three parameters were investigated in the insulin formulations (rapid, NPH/isophane and mixed insulins): i) potency determination by liquid chromatographic method (HPLC), which is the gold standard method for insulin stability assessment according to major pharmacopeias, ii) biophysical assessment of the secondary structure of insulin monomers by circular dichroism (CD), iii) residual bioactivity measuring insulin efficiency on insulin receptor (IR) and Akt phosphorylation in two different hepatocytes cell lines.

### Studied samples and tested conditions based on study design

#### Reference samples

A complete series of vials or cartridges of the insulin formulations were stored at 4°C, and used as references for all three analytical methods, namely HPLC calibration curves preparation at each time point of the stability study, circular dichroism (CD) measurements and biological activity assessment.

#### Samples

Three independent series of vials or cartridges were placed in an incubator (Memmert, IPP-400 type). Temperature cycles were programmed as follows: start at 25°C, increase to 37°C in 12 hours, then back to 25°C in 12 hours, in continuous loop mode. These conditions were intended to mimic the field oscillating temperature cycling both in terms of frequency and amplitude. Temperature variation over time in the incubator was monitored using a Tiny Tag data logger (TinyTag Ultra 2, Gemini Data Loggers) (S1 Fig in additional material section). Three aliquots were withdrawn at the defined time points throughout the study and analyzed in triplicate. According to the guidelines of Société Française des Sciences et Techniques Pharmaceutiques (which are based on various guidelines including ICH, ISO 5725 and ISO 17025 documents, U.S. Food and Drug Administration, Eurachem, Pharmacopeial Forum of US and European Commission), 3 replicates are sufficient for performing a method validation. In our case, a total number of 9 results (n = 9, composed of 3 biological replicates analysed 3 times, i.e. 3 technical replicates) were to be interpreted per formulation per time point.

A second series of samples including vials or cartridges of each tested formulation was submitted to temperature cycles, and insulin was taken out daily to simulate a typical daily treatment with dosages of 10 IU in the morning and 12 IU in the evening. A total of 220μl per day of insulin was withdrawn from the vials or cartridges with 1 IU per 10μl. This was intended to assess whether the variation of air volume present in the vials has any influence on the overall stability, since the interface between air and insulin solution has been reported to trigger aggregation [24]. For this series, n = 6 injections at each time point were performed.

A third series of samples was exposed to standard isothermal conditions to allow comparison with previously released studies. Since the median value of temperature during the cycling process remains above the temperature of 31°C indicated by the manufacturers, a series of vials and cartridges was exposed continuously to this temperature, and insulin quantification was performed after 1, 4 and 8 weeks of this treatment. Finally, the two insulin formulations considered as essential medicines by WHO (rapid and NPH/isophane insulins) were exposed to a continuous temperature of 37°C. For each condition, 6 injections were performed.

Another factor detrimental to protein stability is shaking [24], but it was not assessed in this study since insulin vials are usually stored under static conditions at the patients’ homes.

### Methods

#### Insulin quantification and potency determination by liquid chromatographic method (HPLC)

According to pharmacopeias, reversed-phase high performance liquid chromatography (HPLC) coupled with UV detection at 214 or 280 nm is the method of choice for insulin quantification [17,19,21,25–30] and for potency determination because of the correlation that has been described between HPLC quantification and biological activity [17–19,21]. Insulin potency determination by HPLC was verified to be accurate by comparison with the traditional in vivo rabbit biopotency assay [31]. Furthermore, the literature reports that all inactive forms of insulin are totally insoluble [19,32,33] and thus eliminated during sample preparation.

A liquid chromatographic separation method based on a HPLC Waters X-Bridge C18 column (100 x 2.1 mm, 3.5μm, 100 A) maintained at 40°C was developed and validated. The validation step clearly showed that the method is specific, repeatable with Cv values smaller than 0.23% and the necessary linearity in the study concentration range is warranted by the excellent correlation coefficient value (r^2^ = 0.9999) (see additional material section, S2 Fig for a representative calibration curve).

Mobile phase A consists of water + 0.1% trifluoroacetic acid (TFA). Mobile phase B consists of acetonitrile + 0.1%TFA. Elution is programmed as follows: mobile phase B starts from 20% up to 50% in 2 min, stays at 50% B for 0.2 min, then returns to initial conditions in 0.1 min and equilibration step for 1.7 min. The mobile phase flow-rate is set at 0.5 ml/min, and the total run time is 4 minutes.

UV detection wavelength is set at 280 nm (and 214 nm for control reasons). The literature mentions a potential issue of artifacts generated during analysis at acidic pH [33], but the rapidity of the developed method guarantees that no degradation or change in the samples occurs.

The same HPLC method was used for preservatives quantification, since all components of the samples are baseline separated.

#### Sample preparation step, calibration and controls for HPLC-UV analysis

The sample preparation step generally involves an acidic clarification process [25], whose purpose is to break the insulin-Zn complexes in the formulations. This is particularly useful in the case of suspension analyses, since solid particles cannot be injected in the chromatographic system [21]. It must also be noted that some bioactive forms of modified insulin are soluble under acidic conditions, while lack of solubilization after acidification has been correlated to insulin that is not bioavailable [19]. The impact of the acidic clarification procedure as well as the influence of a filtration step on insulin quantification were verified. No significant difference in insulin quantification could be detected. Thus, the sample preparation process allows avoiding the generation of artifacts, since no precipitated insulin species was re-dissolved.

Formulations stored in the cold room (2–8°C) were used to prepare the calibration curve standards. Aliquots of each formulation were simply diluted with the appropriate volumes of MilliQ water to obtain insulin concentrations ranging from 1 to 10 IU/ml. For acidic clarification, 4 μl of 6M HCl per ml of solution were added. All the measured values for T = 0 (n = 6) were considered 100% and were used as references for the whole stability study. At each defined time point, a fresh calibration curve using the reference formulations was prepared according to this procedure, and all quantification values were expressed versus the values obtained at T = 0. All such fresh calibration curves were found perfectly superimposable over a period of 12 weeks, thus indicating that insulin remained at 100% in the formulations used as references and stored at 2–8°C during the whole study (see additional material section, S3 Fig, superimposition of all calibration curves during the whole study, one insulin type shown as example).

To confirm the ability of the developed method to detect any degradation leading to a decrease in insulin quantity, samples were generated in which instability was forced using high temperature and short exposure to shaking. Temperatures higher than the melting temperature of insulin-Zn complexes (reported to be 69.8°C [11]) were chosen because it is expected that insoluble degradation products such as aggregates or fibrils would form. Therefore, an aliquot of all formulations was heated at 80°C under agitation for 30 min, and the resulting samples were analyzed using the developed chromatographic conditions and sample preparation procedure.

#### Circular dichroism (CD)

Near- and far-UV spectra were obtained on a Jasco J-815 circular dichroism spectrophotometer (Jasco, Tokyo, Japan). The samples were scanned in a 0.05 cm cell from 190 to 260 nm (far-UV) or a 10 mm cell from 250 to 320 nm (near-UV), using a bandwidth of 1.0 nm, a response time of 1 s, a data pitch of 0.2 nm, and a scanning speed of 100 nm/min. A Peltier element (Jasco PFD-350S) was used to maintain temperature at 25°C during scans. Each spectrum is an accumulation of 3 scans. Formulations aliquots were diluted in MilliQ water, and the suspension was clarified by addition of HCl 6M. The final insulin concentration was determined by calculation and was 3.383 x 10−^5^ mol/L for far-UV samples, and 3.021 x 10^−4^ mol/L for near-UV samples.

To produce negative control for the bioactivity and CD assays, and based on the 80°C results, it was decided to force the degradation of the samples by heating them at 115°C during 30 min. These samples were also analyzed in terms of insulin quantity.

#### Bioactivity testing

The bioactivity of the stored insulin samples has been assessed using two hepatocytes cellular models, in which insulin efficiency on insulin receptor (IR) and Akt phosphorylation was detected and quantified as previously reported [34]. Briefly, starved cells were stimulated with 10^-8^M insulin for 15 min at 37°C and then immediately frozen by immersion in liquid nitrogen to stop insulin signaling. Cells were then lysed in ice-cold RIPA buffer and equal amounts of proteins were resolved by 5–20% gradient SDS-PAGE followed by Western blotting onto nitrocellulose membranes for immunodetection of insulin receptor and Akt phosphorylation. Constitutive expression and phosphorylation of proteins of interest were detected with specific primary antibodies and HRP-conjugated secondary antibodies using chemoluminescence. Quantifications were performed using the PXi gel imaging system and the Genesys/Genetools softwares (Syngene, Cambridge, UK).

#### Visual inspection

Visual inspection was performed according to European Pharmacopeia prescriptions. For solutions, a visual detection of any color change or variation in clarity was performed in front of a white and of a black surface. For suspensions, the visual inspection included monitoring of appearance of large aggregates (clumps) or material adhering to the cartridge/vial walls (frosting).

## Results

### Insulin quantification by liquid chromatographic method (HPLC) detects insulin instability if present

The analysis of the samples in which instability was forced by high temperature (80°C) showed a decrease in insulin content of 9 to 14% compared to corresponding reference formulations stored at 2–8°C (see S2 Table in Additional material section for details). This is in line with the findings of a previous experiment (heating at 75°C during 1 hour in a water bath), that allowed the authors to link the reduction of the content to the formation of a small amount of dimers detected by SEC analysis [35].

The quantity of insulin detected in the samples heated at 115°C during 30 min for bioactivity and CD measurements, was 85.5%. The sample also appeared yellowish and contained visible structured aggregates. The decrease in insulin concentration observed in the present study is consistent with previously reported observations [35].

### Insulin quantification by HPLC indicates that the insulin formulations stored under oscillating conditions remain stable over a period of 4 weeks

#### T = 0 characterization and quantification

All insulin formulation vials were visually inspected, and no visible alteration was observed. Insulin was also quantified against freshly prepared calibration curves (one for each formulation type tested), and all quantification results were determined to have no significantly difference from the value indicated on the packages (values measured were between 98.0 and 99.9%, n = 9). Confidence intervals ranged from 0.4 to 1.2%, confirming the precision of the method.

#### T = 4 weeks of temperature cycling: Characterization and quantification

None of the examined formulations presented any visual change in terms of yellowish color or larger aggregate formation.

**Fig 2:**
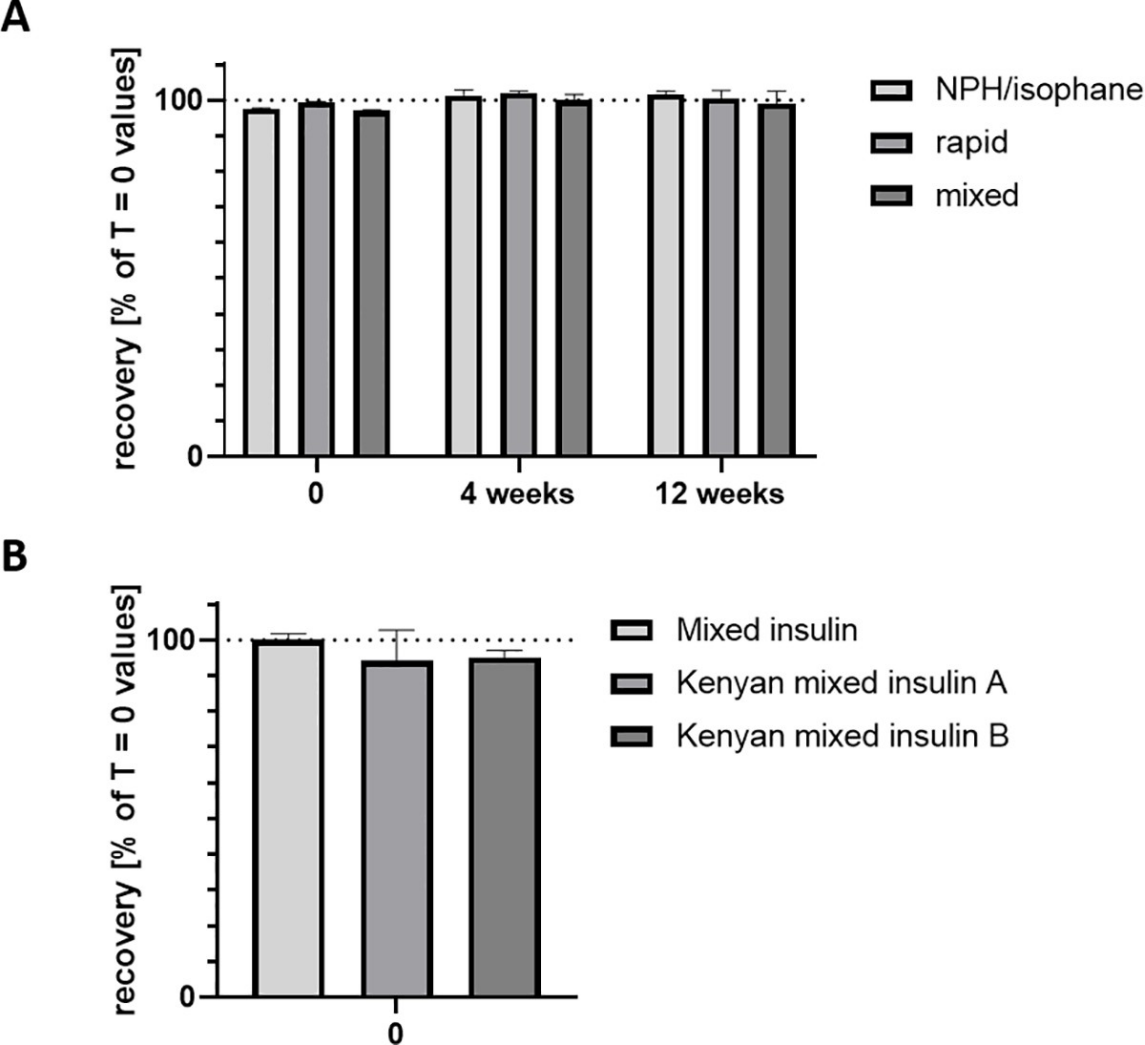
A. HPLC potency determination at T = 0 and after 4 and 12 weeks of continuous temperature cycling (25 to 37°C, conditions reproduced in the laboratory) of the three human insulin formulations used for outpatient management on the field by MSF (NPH/isophane, rapid and mixed insulin). Results are expressed as percentages of concentration determined at T = 0 (n = 9). This graph is representative of the situation for all studied formulations, for which the data is reported in Table 1. B: Comparison between HPLC potency determination of the same mixed insulin as in Fig 2A exposed to 4 weeks of continuous temperature cycling and two samples of mixed insulin that came back from Kenya after 4 weeks of storage at ambient temperature at a patient’s home. Results are expressed as percentages of insulin concentration of the reference kept at 2–8°C (mixed insulin; n = 9).

The measured values of insulin quantification in the studied formulations ranged between 98.3 and 99.9% of the T = 0 concentrations (n = 9, see Fig 2A). These values are all within the pharmacopeia acceptable range (100% +/- 10%).

**Table 1:** Recoveries of insulin at T = 4 and 12 weeks of temperature cycling, expressed as percentages of values measured at T = 0.

Insulin type	T 4 week Recovery [% of T0 value]	T 12 week Recovery [% of T0 value]
Lispro analog	101.7 +/- 5.3	99.4 +/- 0.7
Mixed lispro analog	103.3 +/- 2.5	102.7 +/- 6.1
Glargine analog	98.4 +/-2.4	99.8 +/- 1.0
Aspart analog	99.7 +/- 1.6	100.2 +/- 0.9
**NPH isophane**	**101.3 +/- 1.3**	**101.6 +/- 8.9**
**Rapid**	**100.1 +/- 0.5**	**101.3 +/- 2.3**
**Mixed**	**100.6 +/- 1.6**	**101.9 +/- 3.5**

(in bold characters: the three human insulins used on the field).

#### Later time points

Based on these observations, the decision was taken to leave the samples under temperature cycling conditions for a further duration, up to 12 weeks total. These periods of time largely exceed the “in-use” period suggested by the manufacturers, which is generally four or six weeks after opening the vials, but this period represented a worst-case scenario.

After 12 weeks of temperature cycling, the determined values for the studied formulations were still between 96.4 and 101.9% of T = 0 measured concentrations (n = 9, see Fig 2A).

Fig 2A shows the stability of mixed insulin (which is one of the insulins used for outpatient management on the field by MSF) and the two human insulins recognized as essential medicines by WHO (NPH/isophane and rapid insulin) at 4 and 12 weeks of temperature cycling. Fig 2B includes vials brought back from Kenya to Geneva after 4 weeks of storage and use at ambient temperature at a patient’s home.

#### Influence of air volume variation in the vial

Insulin was withdrawn daily from the study samples in order to investigate the impact of air volume variation, and quantification of the insulin concentration indicated no significant influence on insulin stability under the study conditions. For a period of four weeks, the insulin concentration remained between 97.6 and 101.6% (expressed as a percentage of the values obtained for T = 0; see S3 Table in Additional material for details).

#### Isothermal studies at 31 and 37°C

While storing and studying insulin under oscillating temperatures is innovative, more classical isothermal storage experiments were also performed with the same insulin formulations and compared with data from the literature as well as from the studied oscillating temperatures.

Since the median value of temperature during the cycling process (31°C) remains above the temperature specified by manufacturers, a series of vials was exposed continuously to this temperature, and insulin quantification was performed at 1, 4 and 8 weeks.

All insulin formulations started to degrade after 4 weeks (degradation proportion between 5 and 13.5%, compared to values obtained for the corresponding formulations at T = 0). There was further degradation after eight weeks (12 to 19%), with all formulations out of the range accepted by the pharmacopeia (100 +/- 10%) (see S4 Table in Additional material section).

The two insulin formulations considered as essential medicines by WHO were exposed to a continuous temperature of 37°C. Both were determined to be out of range after one week for the suspension (15.4% decrease) and two weeks for the solution (12.5% decrease). These results are in agreement with previously released data on insulin stability kept at higher isothermal temperature [21].

All together, these data clearly show that the used protocol is able to detect degradation in a quantitative manner and confirm what has been observed in previous insulin stability studies [21], namely that insulin formulations stored isothermally at temperatures higher than ambient temperatures (25–30°C) degrade rapidly and no longer conform with pharmacopeia after 4 weeks.

#### Antimicrobial protection is maintained throughout the study

In order to confirm antimicrobial protection, quantification of the preservatives present in the formulations was performed by HPLC at T = 0 and after the temperature cycling period (see S4 Fig in Additional material section) to determine whether degradation and/or decrease in concentration and therefore in efficacy occurred.

After 12 weeks of continuous temperature cycling, concentrations of the preservatives phenol and metacresol were 92.4 to 94% and 92.7 to 97.7% of claimed values, respectively. This indicates proper antimicrobial activity in the formulations. Furthermore, we did not detect any microbial contamination while taking TEM images of the same samples (S5 Fig in Additional material section).

#### The 3D conformation of insulin is conserved under oscillating storage conditions

**Fig 3:**
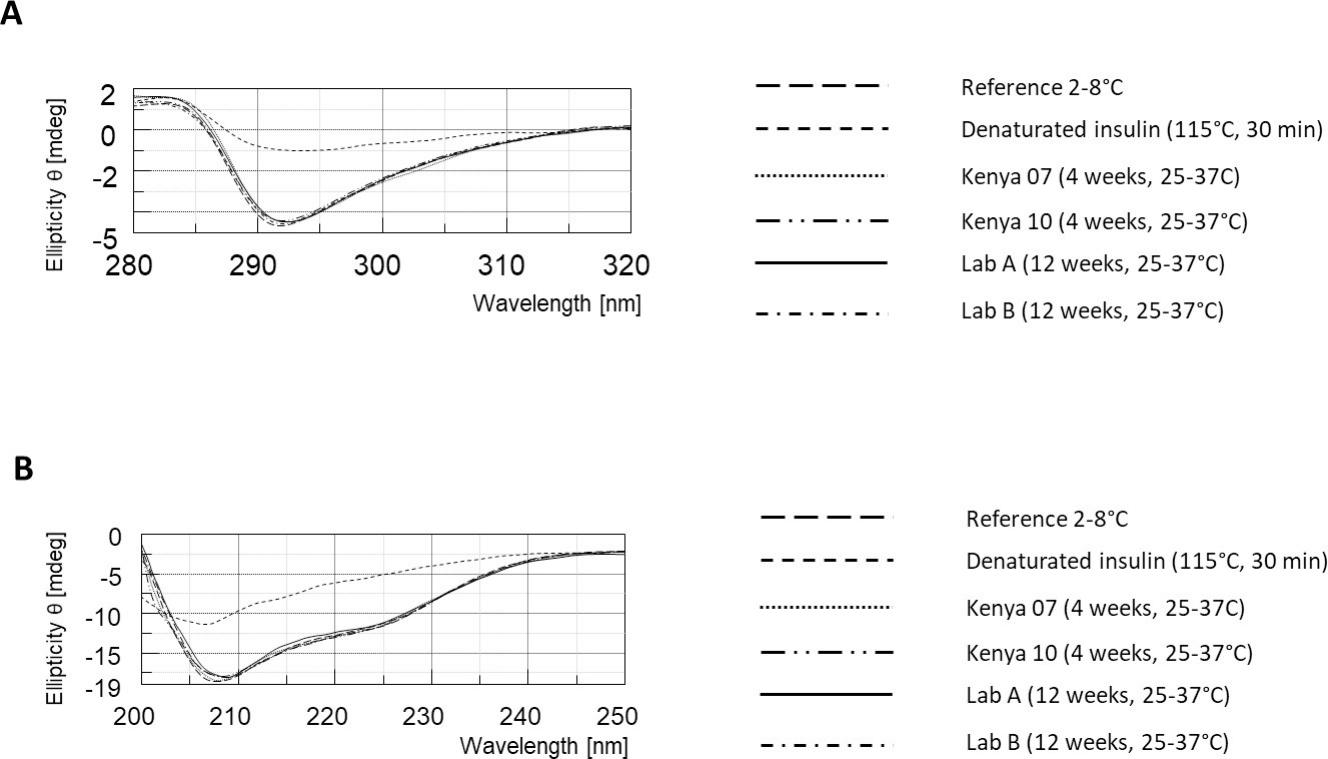
CD of insulin samples. A) Near-UV measurement B) Far-UV measurement. The final insulin concentration was determined by calculation and was 3.383 x 10−^5^ mol/L for far-UV samples, and 3.021 x 10^−4^ mol/L for near-UV samples.

Near UV and far UV CD spectra of the same mixed insulin samples as above were measured. Fig 3 displays the overlay of spectra obtained for reference insulin stored at 4°C, two independent samples of insulin having undergone 25 to 37°C temperature variations for 12 weeks, two samples of mixed insulin from Kenya after 4 weeks at ambient temperature at a patient’s home, and a denatured sample of insulin (heated at 115°C during 30 min). As can be seen, tridimensional conformation of insulin monomers is identical in all the investigated samples, except in denatured mixed insulin. This observation is in agreement with the bioactivity assessment results (see below).

#### Insulin bioactivity is not significantly altered under oscillating storage temperatures

Residual bioactivity of mixed insulin was verified on two hepatic cell lines for insulin samples that were exposed to fluctuating temperatures (25–37°C) for 12 weeks and for a reference formulation stored at 2–8°C (values are mean +/- SEM of 4 different experiments).

**Fig 4:**
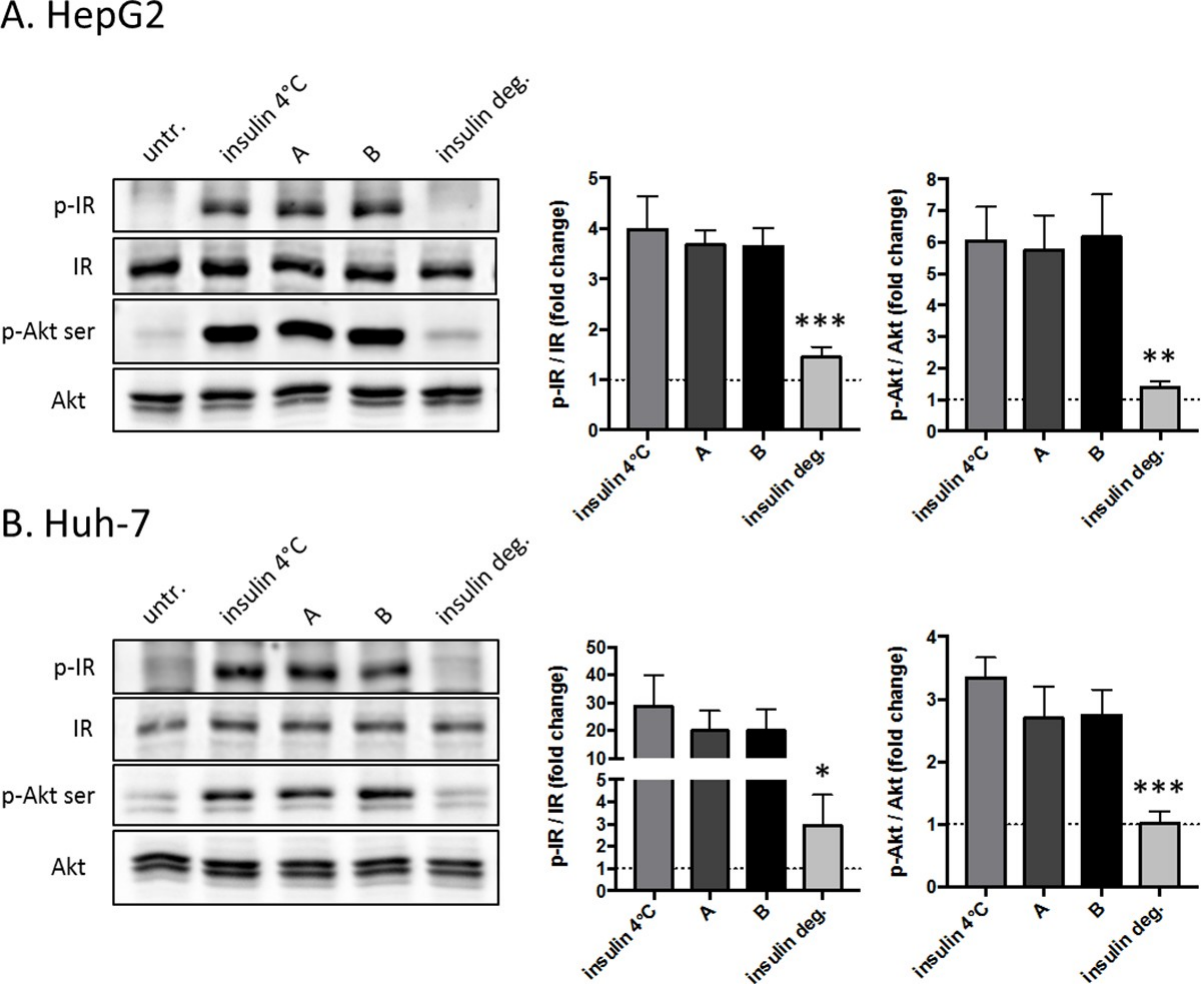
Efficiency of mixed insulin on insulin receptor (IR) and Akt phosphorylation in hepatocytes. Representative Western blots (left panels) and quantification of phosphorylated over total protein levels of insulin receptors and Akt (right panels) in hepatic HepG2 (A) and Huh-7 (B) cells. After overnight serum starvation, cells were stimulated 15 min at 37°C with 10^-8^M of each different insulin. "Insulin 4°C " is mixed insulin constantly stored at 4°C as recommended by the manufacturer, "A" is mixed insulin kept 4 weeks out of refrigeration in a patient’s home in Kenya,”B" is mixed insulin undergoing T° variations between 25 and 37°C for 12 weeks and”insulin deg." is mixed insulin degraded 30 min at 115°C. Values are mean ± SEM of 4 different experiments. Values were considered significant when compared to insulin 4°C: *p≤ 0.05, **p≤ 0.01 or ***p≤ 0.001.

To that end, insulin-induced phosphorylation of the insulin receptor (IR) and Akt (which are key intracellular signalling pathways activated by insulin to control glucose homeostasis) was assessed in two classical cell models of hepatocytes, i.e. HepG2 and Huh-7 cells. Fig 4 displays representative Western blots (left panels) and quantification of phosphorylated over total protein levels of insulin receptors and Akt (right panels) in hepatic HepG2 (A) and Huh-7 (B) cells. After overnight serum starvation, cells were stimulated for 15 min at 37°C with 10^-8^M of each different insulin.

These data clearly indicate that mixed insulin exposed to conditions A (mixed insulin kept 4 weeks out of refrigeration in a patient’s home in Kenya) and B (mixed insulin undergoing T° variations between 25 and 37°C for 12 weeks) still maintain a bioactivity similar to insulin kept at 4°C ("insulin 4°C "). On the opposite, insulin samples heated at 115°C for 30 minutes (”insulin deg.") showed clearly a strong decrease in their bioactivity. These results are consistent with the potency determination and circular dichroism results obtained for the same samples.

## Discussion

The study presented here aimed at assessing the stability of insulin during its period of use by patients in regions presenting oscillating ambient temperatures which vary from those recommended by manufacturers. This study is focused on the usual 28 days of patient use of insulin, and it is clear that the storage conditions of insulin at the healthcare professional level should be maintained according to current recommendations, i.e. adequate cold chain management from manufacturing until the point of delivery to patients.

In insulin formulations, zinc ions are added to stabilize insulin molecules as hexameric complexes [13,24,36], thus hiding hydrophobic parts in the middle of these structures and preventing self-aggregation during storage [24,37]. Phenolic conservative agents also seem to have a stabilizing effect on insulin molecules’ aggregation process [24]. Under destabilizing conditions, such as agitation or heating [12,37–40], the hexamer dissociates. The released monomers, after unfolding, are very prone to form fibrils [32,38,39]. These may cause many medical and biotechnological challenges in terms of production, storage and delivery of soluble insulin (via insulin pumps), thus leading to reduction in efficacy correlated with poor therapeutic results [36]. They may eventually represent an issue in terms of cytotoxic effects [39]. Monitoring protein aggregation is important for the clinical use of biopharmaceutics because aggregated forms of proteins are usually more toxic than the native form [14,24], particularly when aggregates are amyloids. The amyloid fibrils formed [41] are insoluble in most aqueous solutions [19] and biologically inactive. The formation of insulin polymers seems to be five to ten times faster at 37°C than at 25°C [14].

Furthermore, polymeric forms of insulin may also present immunogenic properties [14,24,33,35], as confirmed by detecting specific IgG antibodies against covalent insulin dimers in 30% of insulin-treated diabetic patients [24]. The formation of amyloid aggregates *in situ* when daily injections are performed in the same place may also occur occasionally in the body [42,43]. Such inhomogeneity may promote greater variability in terms of dose delivery precision and consequent therapeutic efficacy [24]. In addition, aggregation or precipitation of the insulin may induce occlusion of injection material [32]. It should be noted that intermediate soluble entities formed during the process were determined to retain native activity and are not associated with adverse immunogenicity [14]. To avoid all these possible drawbacks, insulin must retain the right tridimensional structure and the appropriate efficacy during storage and usage.

In this study, we analyzed the quality of insulin formulations stored under different conditions with respect to structure and efficacy.

As noted in many pharmacopeias [17,19,21,25–30], high performance liquid chromatography is considered to be the gold standard for determining insulin potency. A perfect correlation between bioactivity and potency determination by HPLC is mentioned in the literature and was verified by the experiments performed in the present study. Indeed, a perfect correlation between HPLC quantification of insulin, residual bioactivity and tridimensional conformation of insulin monomers was demonstrated, since identical results were observed for cells exposed to insulin stored at 2–8°C and insulin formulations exposed to oscillating temperature (25–37°C, 12 weeks, mixed insulin). The fast HPLC method developed for the present study is a simple, precise and robust way to evaluate insulin stability in pharmaceutical formulations. The very short analysis duration (4 min per run) allows the analysis of many samples in a reasonable period of time, and it reduces both the environmental burden and overall costs of analyses by reducing the amount of solvent used.

In this study, we have shown that commercial insulin formulations can be used in tropical temperature conditions similar to those studied for 4 weeks. All tested formulations remained in the acceptable range of insulin potency defined by pharmacopeias, up to 12 weeks of thermal cycling from 25 to 37°C, highlighting that the manufacturers’ recommendations for insulin storage during the period of use by the patient are quite conservative. Visual inspection, quantification of preservatives, three-dimensional conformation analysis, residual bioactivity assessment on two cell lines and TEM examination confirmed that no significant differences between insulin submitted to temperature cycling and the samples at T = 0 were observed. The presence of air in the vials and multiple septa punctures had no impact on stability, and no significant difference in stability was found between insulin conditioned in pen cartridges and 10 ml glass vials.

There are no general extended controlled temperature conditions (ECTC) for protein-based therapeutics. However, in 2016 WHO published guidelines on the stability evaluation of vaccines for use under extended controlled temperature conditions (ECTC) as annex 5. It considers some deviations relative to controlled temperature chain programs (CTC), such as single exposure to at least 40°C for a minimum of 3 days while keeping potency at the right level [44]. This allows recommendations for the 4-weeks period of use after opening, with indication of maximal acceptable temperature to guide decision-making when exposure to higher temperatures is planned. The results shown here suggest that an ECTC could be formulated for insulin, since a deviation from the strict storage recommendations during a 4-week period of use does not alter its structure or its efficacy, maintaining pharmacopeia conformity.

To our knowledge, the present study is the first to look at the impact of temperature cycling on insulin stability since all stability studies of biopharmaceuticals involving temperature are done under isothermal conditions, rather than oscillating temperatures [43].

Insulin degradation including microfibril formation is energy dependent [13,40] andtendency to fibrillation increases with temperature [24].

Thermal denaturation is a matter of both time and temperature and is a complex, partially reversible process [12]. Increasing the temperature can perturb the native protein conformation, which promotes unfolding of parts of the protein over time [45]. It has been shown that partly unfolded proteins are more prone to aggregation than the native state of the protein [46]. Thermal energy plays a role in surmounting the transition state. This thermally induced denaturation is time dependent and may be reversible for some proteins [45], but if sufficient energy is added to the system (such as application of high temperatures for a long time period), this usually leads to irreversible denaturation because of aggregation. The results obtained when insulin was continuously exposed to higher temperatures (31°C) over eight weeks are in agreement with previously released data on insulin heat stability [21]. In contrast, significant degradation was not observed when the formulations were submitted to temperature cycling. During the temperature cycling process, the energy over time that is brought to the system is most likely not sufficient to lead directly to irreversible aggregation. Thus, a partially reversible unfolding of the protein can be hypothesized, avoiding nucleation and fibril formation. Whereas under continuous heating conditions, the amount of energy may be sufficient to promote irreversible conformational changes of the insulin molecules, leading to a nucleation process followed by fibril elongation. This is important as past publications and current tests on biological products are performed at isothermal temperatures and not using fluctuating temperatures. As such, current protocols should be extended to consider the reality of temperature fluctuations present in most settings where patients live, because this may have a big impact on recommendations for storage of biological products during the period of use.

Of note, this study does not provide data for generalisability beyond the temperature ranges studied, nor information on insulin stability during storage outside of cold chain prior to the period of use by the patient, as cold-chain was ensured until the point of delivery during this study. As such, the need to ensure proper cold chain during transport and pre-patient storage remain as challenges to its use. Furthermore, even the fleeting exposure of insulin to high temperatures may result in the formation of minute concentrations of amyloidogenic seeds, shortening its half-life. Whilst pre-use cold chain integrity was ensured for the insulins used in the study, the conclusions of the study therefore rely on the vials being seed-free at t = 0. This may not be reflected in real-world conditions, where lack of cold chain integrity throughout transport and storage may mean that insulin has been exposed to higher than recommended temperatures even prior to dispensation to the patient.

## Conclusion and perspectives

These findings—showing that insulin retains structural and efficacy integrity over a period of four weeks’ exposure to oscillating temperatures—open new perspectives for diabetes care in regions where strict storage at 2–8°C is not available at patient level. Work is in progress to explore other temperature settings and extrapolate the results to other locations. However, this study already confirms the possibility for health professionals working in similar temperature conditions to give insulin to patients with diabetes, for a period of use of up to four weeks, even in the absence of patient access to refrigeration. This observation suggests a possible use of insulin similar to that recommended by the manufacturers for temperate countries. Many challenges still confront health care providers and patients trying to manage diabetes in low resource settings, but the ability to use insulin without cold chain during the period of use in hot climates removes at least one significant barrier.

## Supporting information

S1 Fig Temperature monitoring curve in the incubator (TinyTag data), reproducing the temperatures fluctuations measured on the field. (TIF)

S2 Fig Typical calibration curve obtained for Humalog insulin applying the developed HPLC conditions. All values are represented on the graph (n = 6 independent injections).
(TIF)

S3 Fig Superimposition of all calibration curves freshly prepared from formulations stored at 2–8°C for each time point of the study. Example of Humalog.
(TIF)

S4 Fig Typical chromatogram of insulin formulation containing two preservatives: Phenol and metacresol, showing no co-elution with insulin molecule. Example of Novorapid.
(TIF)

S5 Fig TEM image of an insulin formulation at T = 0 (left) and at T = 12 weeks of continuous temperature cycling (25–37°C, right). (TIF)

S1 Table Listing of tested formulations, including manufacturers recommendations for storage and handling of insulin formulations. (PDF)

S2 Table Insulin concentration determined after short exposure to high temperature (80°C, 30 min). Values are expressed as percentage of T = 0 determined values.
(PDF)

S3 Table Insulin quantification values for assessing the influence of the air volume variation in the vials during 4 weeks of continuous temperature cycling between 25 and 37°C. Results are expressed as percentage of T = 0 determined values for corresponding formulations.
(PDF)

S4 Table Insulin quantification at T = 1, 4 and 8 weeks of continuous exposure to a temperature of 31°C, which corresponds to the median value of the temperature cycles. Values are expressed as percentage of T = 0 determined values.
(PDF)

S1 Raw images (PDF)
