# Supplementary figures and images for: Heat-stability study of various insulin types in tropical temperature conditions: New insights towards improving diabetes care
Source: PLoS One. 2021 Feb 3;16(2):e0245372. doi: 10.1371/journal.pone.0245372 (PMC7857579; doi:10.1371/journal.pone.0245372)

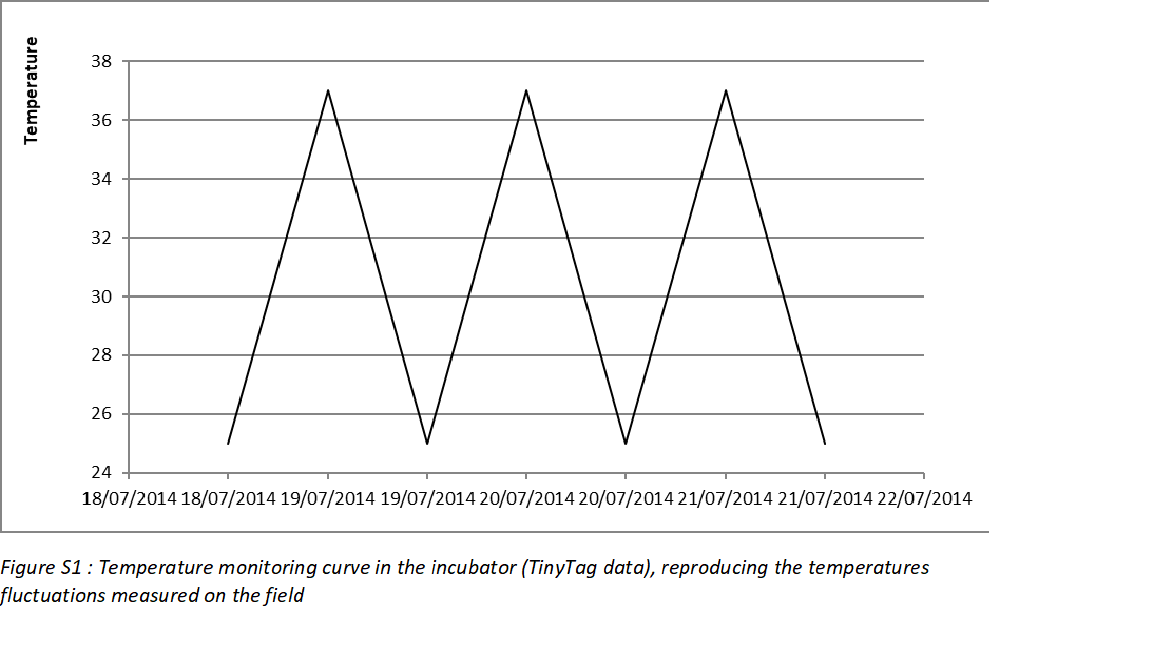

Supplement: S1 Fig — (TIF) [file pone.0245372.s001.tif]

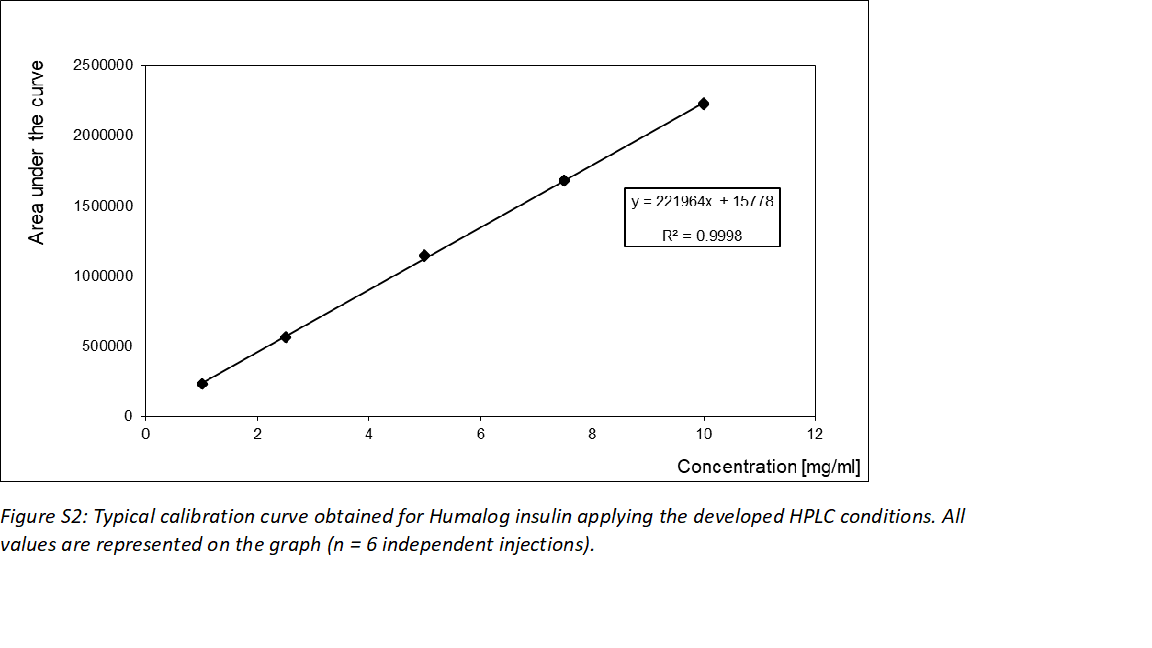

Supplement: S2 Fig — All values are represented on the graph (n = 6 independent injections). (TIF) [file pone.0245372.s002.tif]

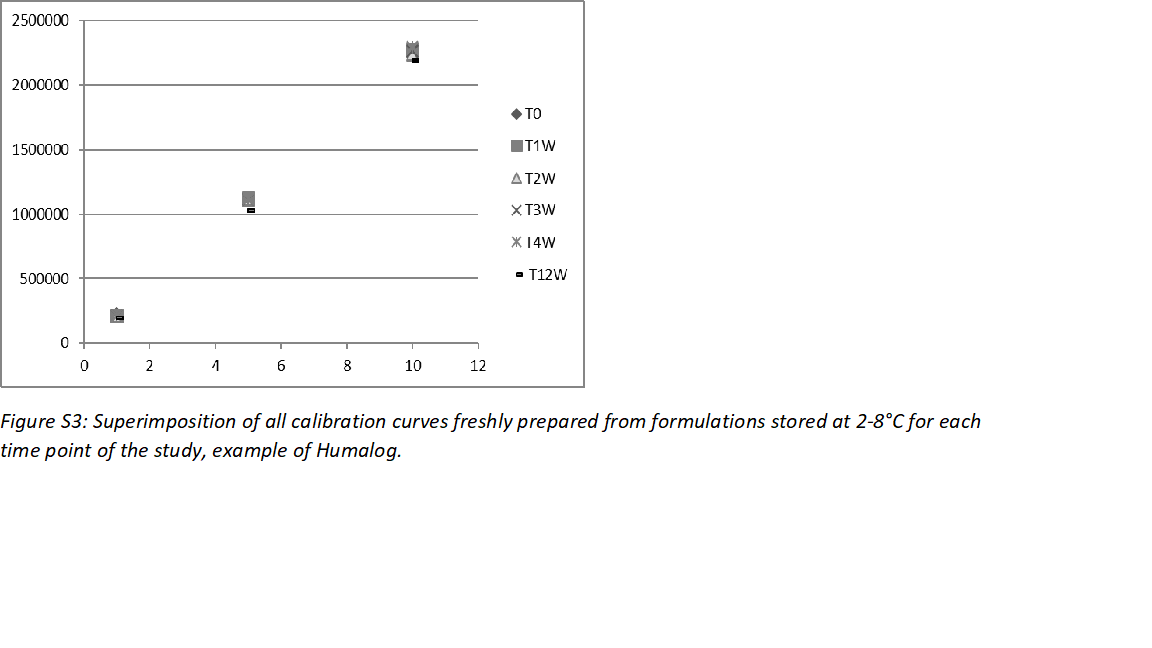

Supplement: S3 Fig — Example of Humalog. (TIF) [file pone.0245372.s003.tif]

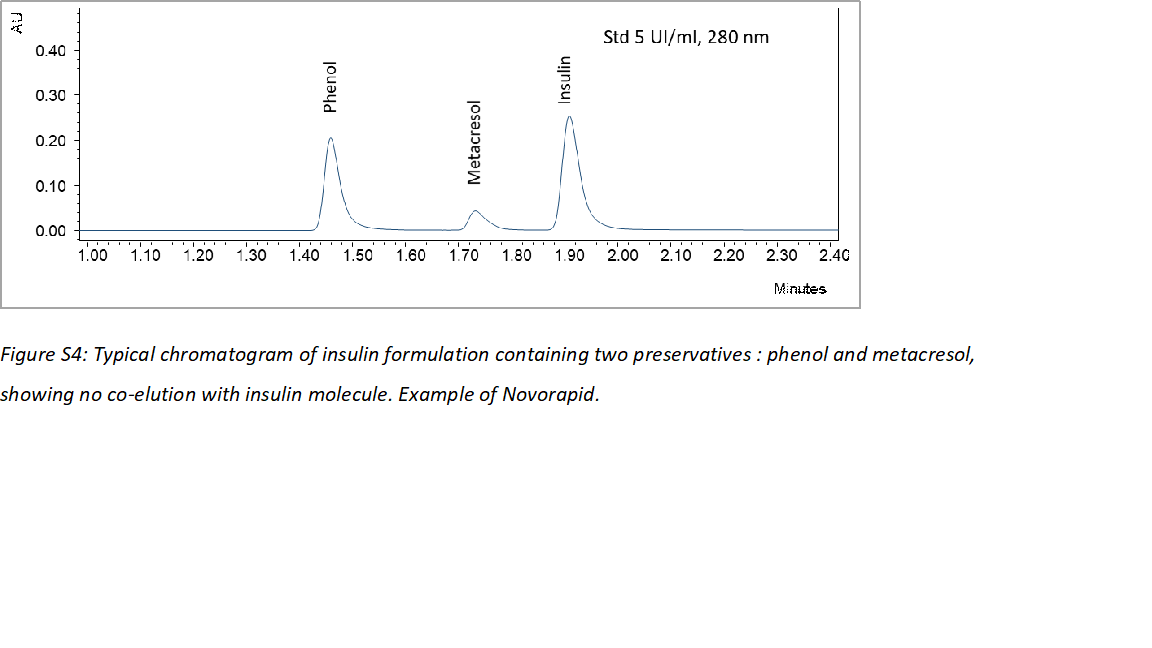

Supplement: S4 Fig — Example of Novorapid. (TIF) [file pone.0245372.s004.tif]

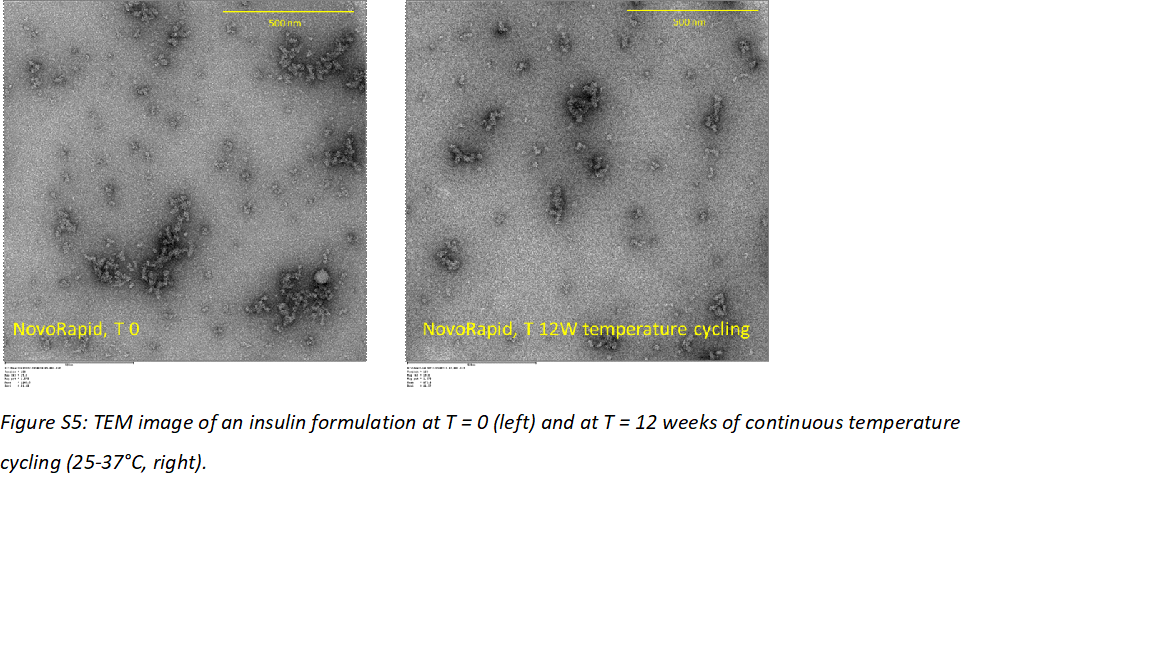

Supplement: S5 Fig — (TIF) [file pone.0245372.s005.tif]
