# Supplementary material for: Heat-stability study of various insulin types in tropical temperature conditions: New insights towards improving diabetes care
Source: PLoS One. 2021 Feb 3;16(2):e0245372. doi: 10.1371/journal.pone.0245372 (PMC7857579; doi:10.1371/journal.pone.0245372)
Supplement: S1 Table — (PDF) [file pone.0245372.s006.pdf]

Table S1: Listing of tested formulations, including manufacturers recommendations for storage and handling of insulin formulations

Insulin type	Conditioning	Storage temp. before opening [°C]	Recommended usage time once open	Maximal temp. during usage	Other storage recommendations
Novorapid 100 IE (Novo Nordisk)	Solution, 3ml prefilled pen	2°C - 8°C	4 weeks	30°C	Do not freeze. Keep the vial in the outer carton in order to protect from light
Insulatard HM 100 IE (Novo Nordisk) *	Suspension, 10 ml flask	2°C - 8°C	6 weeks	25°C	Do not freeze<br>Keep the vial in the outer carton in order to protect from light
Lantus Solostar 100 UI/ml<br>(Sanofi Aventis)	Solution, 3ml prefilled pen	2°C - 8°C	4 weeks	25°C	Do not freeze<br>Protect from light<br>Do not use after 4 weeks at RT (note the opening date on the vial)
Humalog KwikPen 100 IE (Eli Lilly)	Solution, 3ml prefilled pen	2°C - 8°C	4 weeks	15-25°C	Do not freeze<br>Protect from light, avoid direct sunlight exposure;<br>do not use after 4 weeks at RT
Humalog KwikPen mix 25 100 IE<br>(Eli Lilly)	Suspension, 3ml prefilled pen	2°C - 8°C	4 weeks	15-25°C	Do not freeze<br>Protect from light, avoid direct sunlight exposure;<br>do not use after 4 weeks at RT
Actrapid 100 UI/ml (Novo Nordisk) *	Solution, 10ml flask	2°C - 8°C	6 weeks	25°C	Do not freeze<br>Keep the vial in the outer carton in order to protect from light.<br>During usage period, do not put in a fridge or freezer
Mixtard 30<br>(Novo Nordisk)	Suspension, 10ml flask	2°C - 8°C	6 weeks	25°C	Do not freeze<br>Keep the vial in the outer carton in order to protect from light.<br>During usage period, do not put in a fridge or freezer

*Insulins considered as essential medicines by WHO
