# Supplementary material for: Heat-stability study of various insulin types in tropical temperature conditions: New insights towards improving diabetes care
Source: PLoS One. 2021 Feb 3;16(2):e0245372. doi: 10.1371/journal.pone.0245372 (PMC7857579; doi:10.1371/journal.pone.0245372)
Supplement: S2 Table — Values are expressed as percentage of T = 0 determined values. (PDF) [file pone.0245372.s007.pdf]

Table S2: Insulin concentration determined after short exposure to high temperature (80°C, 30 min). Values are expresses as percentage of T = 0 determined values.

Humalog	Humalog Mix25	Lantus	Novorapid	Insulatard HM
87.5 +/- 2.6	86.0 +/- 0.7	86.4+/- 0.7	87.6+/- 1.0	90.8+/- 6.3
