# Supplementary material for: Heat-stability study of various insulin types in tropical temperature conditions: New insights towards improving diabetes care
Source: PLoS One. 2021 Feb 3;16(2):e0245372. doi: 10.1371/journal.pone.0245372 (PMC7857579; doi:10.1371/journal.pone.0245372)
Supplement: S3 Table — Results are expressed as percentage of T = 0 determined values for corresponding formulations. (PDF) [file pone.0245372.s008.pdf]

Table S3: Insulin quantification values for assessing the influence of the air volume variation in the vials during 4 weeks of continuous temperature cycling between 25 and 37°C. Results are expressed as percentage of T = 0 determined values for corresponding formulations.

Sample	Humalog	Humalog Mix25	Lantus	Novorapid	Insulatard HM
Ref. [2-8°C]	99.6 +/- 3.5	99.5 +/- 2.3	100.0 +/- 1.3	100.0 +/- 2.5	100.3 +/- 1.2
4 weeks [25-37°C]	98.8 +/- 0.3	98.3 +/- 2.3	98.5 +/- 2.0	99.9 +/- 1.4	99.2 +/- 13.5
4 weeks [25-37°C, daily dose simulation]	98.5 +/- 1.7	97.6 +/- 3.3	98.2 +/- 1.8	100.0 +/- 2.1	101.6 +/- 1.8
