# Supplementary material for: Heat-stability study of various insulin types in tropical temperature conditions: New insights towards improving diabetes care
Source: PLoS One. 2021 Feb 3;16(2):e0245372. doi: 10.1371/journal.pone.0245372 (PMC7857579; doi:10.1371/journal.pone.0245372)
Supplement: S4 Table — Values are expressed as percentage of T = 0 determined values. (PDF) [file pone.0245372.s009.pdf]

Table S4: Insulin quantification at T = 1, 4 and 8 weeks of continuous exposure to a temperature of 31°C, which corresponds to the median value of the temperature cycles. Values are expressed as percentage of T = 0 determined values.

Time	Humalog	Humalog Mix25	Lantus	Novorapid	Insulatard HM
1 week	101.5 +/- 2.6	100.8 +/- 0.7	97.2 +/- 0.7	98.3 +/- 1.0	100.2 +/- 6.3
4 weeks	91.0 +/- 1.9	87.5 +/- 0.9	86.5 +/- 0.9	93.2 +/- 1.0	94.8 +/- 5.4
8 weeks	84.3 +/- 2.6	80.8 +/- 2.9	84.3 +/- 1.8	87.5 +/- 2.2	82.8 +/- 4.0
