# Supplementary material for: Heat-stability study of various insulin types in tropical temperature conditions: New insights towards improving diabetes care
Source: PLoS One. 2021 Feb 3;16(2):e0245372. doi: 10.1371/journal.pone.0245372 (PMC7857579; doi:10.1371/journal.pone.0245372)
Supplement: S1 Raw images — (PDF) [file pone.0245372.s010.pdf]

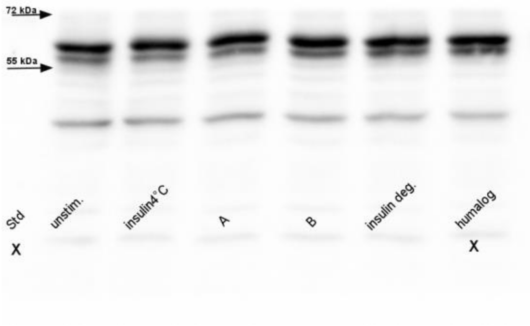

Gel 3c Akt HepG2

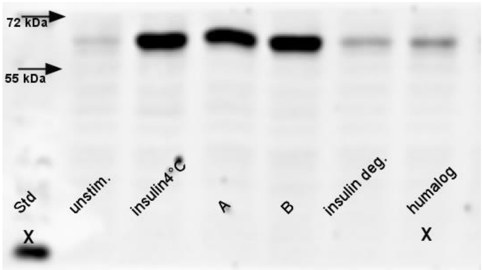

Gel 4c P Akt HepG2

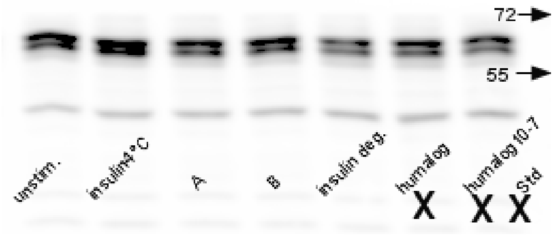

Gel 3c Akt Huh7

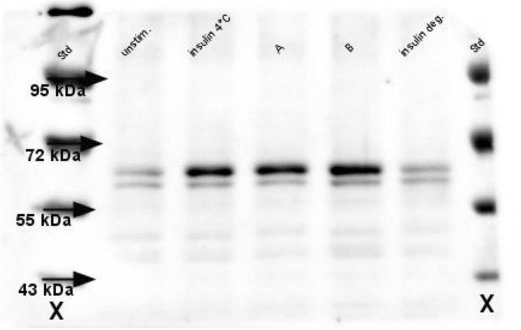

Gel 11 P Akt Huh7

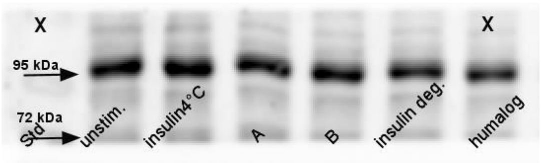

Gel 4b IR HepG2

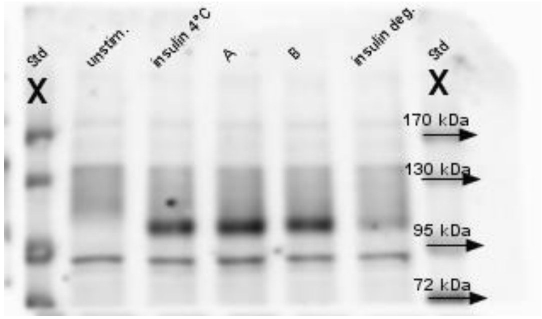

Gel 8a P IR HepG2

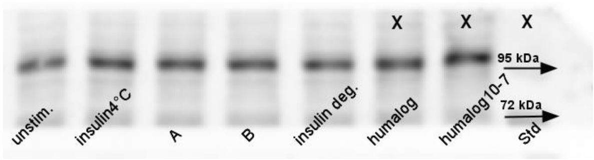

Gel 4b IR Huh7

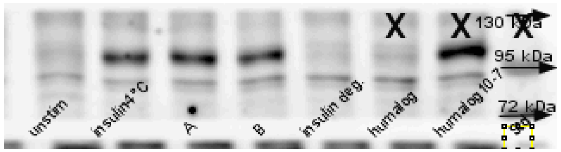

Gel 3b P IR Huh7

Labels:

- X” is linked to any lane which remains unused in the final Figure
- Std = protein ladder
- “unstim” = cell line (either HepG2 or Huh7) with no stimulation using mixed insulin
- “Insulin 4°C " is mixed insulin constantly stored at 4°C as recommended by the manufacturer
- "A" is mixed insulin kept 4 weeks at RT in Kenya, at a patient’s home,
- ”B" is mixed insulin which underwent continuous T° variations between 25 and 37°C for 12 weeks
- ”insulin deg." is mixed insulin degraded 60 min at 115°C
- “Humalog” is cell line stimulated with Humalog insulin 15 min at 10 -8 M (control)
- “Humalog 10-7” is cell line stimulated with Humalog 15 min at 10 -7 M (control)

Methods:

- Detection by luminescence, using ECL select as reagent (Ref. N°: RPN 2235, from Amersham)
- Acquisition software : GeneSys
- System : Pxi6 (Syngene)
- Format : sgd and tiff
- TIFF Images labelling software : ImageJ application Fiji
- Protein standard : Page Ruler Prestained Protein ladder (Ref. N° : 26616, from Thermo scientific)
